# Evolving Food Choices Among the Urban Indian Middle-Class: A Qualitative Study

**DOI:** 10.3389/fnut.2022.844413

**Published:** 2022-03-29

**Authors:** Gargi S. Kumar, Mrinmoyi Kulkarni, Neha Rathi

**Affiliations:** ^1^Department of Humanities and Social Sciences, Indian Institute of Technology Bombay, Mumbai, India; ^2^Department of Community Medicine, Institute of Medical Sciences, Banaras Hindu University, Varanasi, India

**Keywords:** food choices, qualitative research, India, food environment, processed food

## Abstract

One of the leading risk factors for an escalating obesity burden in India is non-nutritious choices. Underpinned by the nutrition transition theory, this qualitative inquiry was designed to understand the urban middle-class Indian consumers’ views about processed foods and rapidly changing food choices. The study consisted of two phases, the first phase consisted of focus group discussions pertaining to the definition and conception of processed foods and the second phase consisted of interviews regarding the changing food environment. A convenience sample of Indian consumers aged 40–65 years were recruited from Mumbai and Kochi to participate in focus group discussions (FGD1 – nine participants and FGD2 – seven participants) and semi-structured face-to-face interviews (*N* = 22). Both discussions and interviews were audio-recorded and transcribed verbatim. Thematic analysis was used to analyze the transcribed data. Features of processed foods mentioned were chemical and physical processing, prolonged shelf life and poor nutritional quality. Factors influencing food choices and consumption of processed foods reported by participants could be categorized into changes in the socio-cultural environment and changes in the food environment. Changes in the socio-cultural environment included globalization and urbanization, long work days and sedentary living, rise in income levels and decrease in household cooking. Changes in the food environment included increased availability and accessibility of processed foods, replacement of traditional Indian diet with Western food, food as indicators of status, food advertisements and convenience. These results are consistent with nutrition transition theory and provide useful direction for public health policies aimed at promoting healthy diets.

## Introduction

Post globalization, India has witnessed a dramatic rise in the prevalence of overweight and obesity among its adult population (1, 2). Luhar et al. have estimated that the prevalence of overweight will more than double, while the prevalence of obesity will triple among Indian adults aged 20–69 years between 2010 and 2040 (2). This unprecedented rise in overweight and obesity has been accompanied by significant increases in the burden of chronic degenerative diseases including diabetes, cardiovascular disease, respiratory illness, and certain forms of carcinomas (3, 4). Indeed, in 2016 these non-communicable diseases (NCDs) accounted for 63% of all deaths in India (5), resulting in compromised quality of life, diminished work productivity, and enormous health care costs. In light of this, it is imperative to understand the risk factors associated with this crisis to inform nutrition and public health policies.

The Centre for Macro Consumer Research predicts that by 2025–2026 the middle class in India will grow from an estimated 53.3 million households (267 million individuals) in 2015–2016 to approximately 113.8 million households (547 million individuals) (6). The growth and consumption habits of the middle class who live in urban areas serve as a useful measurement of how living standards in India are changing. Rapid urbanization and changes in social and cultural practices among the new middle class have altered food habits (7).

A leading risk factor for the increasing disease burden is consumption of processed food (8–11). Since liberalization in the early nineties, there has been a drastic change in food consumption patterns of urban Indians (12–14) characterized by increased consumption of sugars, fats, oil, and ultra-processed foods (12–15). Certainly, there has been a notable increase in the purchase of sweet and salty snacks from 2013 to 2017 in India (16). For example, the per capita annual purchase of sweet snacks was 1.64 kg in 2013 which rose to 1.93 kg (17%) in 2017 (16). In the same vein, a rising trend was observed in per capita purchases of edible oils between 2013 and 2017 (by 0.44 kg, 4%) (16) and subsequently, an increase in oil consumption was reported in urban India (17). Similar shifts in consumption have been reported in other low- and middle-income economies as well (18–20).

Interestingly, a connection between Gross Development Product (GDP) and per capita structural change in diet was seen by Perisse et al. while analyzing the data for 85 countries (21) a finding later confirmed by Drewnowski and Popkin when comparing it with a similar data set from 1993 (22). The salient factors were the increase in energy intake and change in structure of diet – in terms of increased intake of fat. This change in food intake due to the influence of economic factors has been referred to as the “nutrition transition” (22). Elaborating on the nutrition transition, Popkin (23) delineated five stages, the first of the hunter gatherer who ate a variety of wild animals and plants and had low life expectancy, the second stage was the beginning of agriculture with a dominant cereal diet and low life expectancy, in stage three the beginning of industrialization was marked by a variety of foods, decline of famine and slow decline of mortality (20). The fourth stage is characterized by increased intake of fat and sugar leading to obesity and non-communicable disease, stage five is characterized by a decrease in processed food in an attempt to postpone NCDs (23). Apart from structural factors (e.g., GDP and energy needs), individual level factors such as food choice also need to be studied to map the nutrition transition in different cultures.

Food choice decisions are described as being recurrent, multifaceted, contextual, dynamic, and complex which result in different forms of food behaviors (24). Adopting different theoretical frameworks, previous reviews of food choice have identified three key determinants affecting them, which include environmental (e.g., physical surroundings, type of food presentation and location, time-related characteristics) (25, 26), social (e.g., social modeling and social norms) (27), and psychological factors (e.g., past behavior, habit strength, hedonic appreciation, and motivational regulation) (28, 29). Scholars further note that the final outcome of food choice is based on the interactions between these three factors (30–32). Nevertheless, Sobal and Bisogni (24) argue that no single theory or model could comprehensively explain the complex food choice decision making phenomenon and therefore recommend the need for multiple perspectives including the development of new food choice models informed by constructionist thinking (24).

Traditional Indian cuisine was determined by regional and caste factors (33), however, with liberalization and urbanization, global elements are now reflected in the Indian diet. A preference for animal products, temperate zone fruits, processed convenience foods and drinks is seen (34). A preference for wheat is also seen among south Indians who were traditionally rice eaters (34). Consumers are no longer limited to local produce but literally have the world at their fingertips. Processed and ultra-processed foods have emerged as an increasingly popular food choice in contemporary urban societies (16, 18, 35, 36). Processed foods as described by NOVA food classification, are food items prepared by adding salt, sugar, oil, or other processed culinary ingredients to unprocessed or minimally processed foods employing preservation techniques like canning and non-alcoholic fermentation (37). Canned vegetables, canned fish and salted nuts are few examples of commonly consumed processed foods (37). While ultra-processed foods are food and beverage formulations prepared mostly or completely from substances extracted from food or derived from food constituents, which finally undergo a series of industrial processes before reaching the consumer (37). Biscuits, carbonated beverages, canned fish, ready-to-eat meals, powdered soups, instant noodles are some typical examples of ultra-processed foods (37). The burgeoning popularity of these ultra-processed foods is attributed to its easy accessibility and availability, cheap price, convenience, hyper-palatability, and ubiquitous marketing (35, 38, 39). Owing to these attractive features, ultra-processed foods have gradually begun displacing home-cooked meals and fresh fruits and vegetables in traditional diets (35–37). Moreover, the availability of ultra-processed foods has also been associated with waning household culinary practices and increased snacking episodes as reported in the literature (37, 40–42). The excessive intake of ultra-processed foods has been associated with poor diet quality, increased risk of obesity and NCDs, and premature mortality (37).

Amidst the on-going nutrition transition and escalating NCD burden, there is a dearth of literature focusing on individual perspectives of the urban, middle-class Indians toward the rapid evolution of unhealthy food choices. Therefore, an in-depth study using both focus group discussions and semi-structured face-to-face interviews was undertaken to understand:

(i) How urban Indian middle-class consumers define and perceive processed food.
(ii) Changes in food choice as well as explore the reasons behind it. The reasons are analyzed in terms of a changing socio-cultural as well as food environment perspective.

## Materials and Methods

The study was conducted in two phases, in phase I two focus group discussions were conducted in two Indian cities, Mumbai and Kochi. The focus group discussions served as an exploratory study to throw light on their understanding of processed foods and factors related to its consumption. These results provided the basis for the development of phase II of the study. Phase II consisted of semi-structured interviews that were conducted in Mumbai and Kochi regarding current food habits and perceived changes in eating behavior. These questions were also developed within the framework of the Nutrition Transition Theory (22). Both the phases of this qualitative inquiry have been conducted and reported according to the Consolidated Criteria for Reporting Qualitative Research (COREQ) guidelines (43).

### Phase I (Focus Group Discussions)

#### Study Design

The social constructivist paradigm was employed in this qualitative analysis (44). In this interpretive framework, individuals seek to understand their world and develop their own views that correspond to their experiences (45). These perceptions are typically governed by the surroundings in which the individuals live and are developed through their interactions with the members of the surroundings (45). In addition, this paradigm acknowledges truth and knowledge as being generated by the interactions of individuals within a community (46). Therefore, the use of social constructivist framework in this present context was considered appropriate, as it assisted the researchers in understanding how Indian consumers conceptualize processed foods.

#### Study Setting

Mumbai is a diverse city (i.e., it is home to people from all states of India), where many cuisines have been adopted and transformed into one, unique to Mumbai. Post globalization, Kerala has witnessed an unprecedented consumption boom and increased standard of living (47). Kochi, is an emerging metropolitan city that represents Kerala. The more urbanized states indicated a greater increase in NCDs and obesity over the last decade (48). Both Maharashtra and Kerala fall in the category of urbanized states and hence, the choice of cities like Mumbai and Kochi was justified in representing urban India with respect to change in food choice.

#### Sample and Recruitment

Urban, middle-class consumers aged 40–65 years formed the study sample. Potential participants were recruited from two cities, i.e., Mumbai in the state of Maharashtra and Kochi in the state of Kerala through convenience sampling. Convenience sampling is a type of non-probability or non-random sampling where members of the target population that meet certain practical criteria, such as easy accessibility, geographical proximity, availability at a given time, or the willingness to participate are included for the purpose of the study (49).

Participants in the age group of 40–65 years were recruited for this study because they are vulnerable to the onset of NCDs (50). Individuals’ whose annual income was above 200,000 (50), who are college educated and in white collar jobs were classified as middle-class (51). Participants with a medical or nutritional background were excluded from the study (i.e., both Phase I and II) to avoid any potential bias in the responses.

The investigator contacted participants over the phone and an appointment for group discussion was made as per their convenience. The research protocol for this qualitative investigation (i.e., both Phase I and II) was approved by the Institutional Ethics Committee of the Indian Institute of Technology Bombay (IITB-IEC/2019/029).

#### Data Collection

An focus group discussion (FGD) is an organized open discussion to elicit multiple views about a specific topic (52). Hence, FGD was identified as a suitable data collection technique to understand Indian consumers’ perspectives regarding processed food. Before the commencement of the discussion, written informed consent was taken from all the participants. The participants were verbally notified that the discussions would be audio-recorded and complete confidentiality would be maintained. Two FGDs were conducted, i.e., one in Mumbai (FGD1 – nine participants) and the other one in Kochi (FGD2 – seven participants). FGD1 was carried out in English while FGD2 was carried out in Malayalam (language spoken in Kochi). The typical size of a focus group discussion is 6–12 participants (53). All the participants in FGD1 and 2 actively participated in the discussion. GK being fluent in both the languages, i.e., English and Malayalam facilitated the discussions while a notetaker was appointed to take field notes. An open terrace at one of the participant’s house in both cities served as a venue for the FGDs. Both the FGDs lasted for about 90 min.

Based on the research aim, four open-ended questions (Table 1) were designed to guide the discussions. The questions were framed based on the pilot study and the previous literature pertaining to conceptualization of processed food (54).

**TABLE 1:** Questions posed to the participants during the focus group discussions (FGDs).

Q1	What do you understand by processed foods?
Q2	What factors contributed to the presence of processed foods in the market? Please elaborate.
Q3	What is your perception about the use of processed food?
Q4	What are the pros and cons of consuming processed food?

#### Data Analysis

To ensure consistency and accuracy, the interviewer (GK) herself undertook the transcription and translation of the narrative data. The transcribed data were analyzed thematically using the NVivo 12 software program along with manual coding. This software program facilitated the identification of themes and the extraction of associated quotations from the data set. GK repeatedly read the transcripts to familiarize herself with the breadth and depth of content as well as searched for meanings and patterns emerging from the transcribed information (55). In order to analyze the data, a four-step method which includes data immersion, coding, developing categories, and the classification of themes was employed (55). Themes were developed and thereafter, descriptive quotes were extracted from the transcripts to illustrate the elements of the emerging themes. The remaining two authors (NR and MK) independently analyzed all the transcripts to confirm inter-rater reliability (56). Any difference in opinion was resolved through discussion (45).

### Phase II (Semi-Structured Interviews)

#### Study Design

Like Phase I, social constructivist framework (45, 46) was used to guide the second phase of this qualitative study. Adopting this framework, the study investigators aimed to examine Indian consumers’ perception toward rapidly changing food choice behaviors.

#### Sample and Recruitment

Twenty- two urban, middle-class Indian consumers aged 40–65 years formed the study sample. For individual interviews, sample size can vary from 20 to 30 (57). However, the interviewer (GK) stopped recruiting the respondents after thematic saturation, the point at which no new concepts emerge from subsequent interviews, was achieved (58).

In line with Phase I, the sample was recruited from Mumbai and Kochi through convenience sampling. Potential informants were identified through professional networks of the lead author (GK). GK approached the participants in person to provide an overall description of the study as well as handed over the consent form to the interested participants. Upon receipt of the signed consent forms, GK confirmed the preferred date, time, and location for the interview with the participants.

#### Data Collection

In Phase II, semi-structured face-to-face interviews were used as a data collection method to investigate the changes in food choice behavior among urban middle-class Indians. At the beginning of the interviews, the lead researcher (GK) asked for permission to audio record the conversation and the interviewees were ensured that collected data will be treated as confidential. Based on participants preference, the interviews were either conducted in English or Malayalam and field notes were taken during the interview sessions. Each interview lasted for about 45 min to an hour. All the interviews were conducted in a private room of the interviewees’ homes by the lead researcher (GK). Informants were recruited until the interview data showed saturation of content, the stage when no new ideas were reported. Saturation was reached at 9th (Kochi) and 13th (Mumbai) interviews, respectively, and therefore no more participants were recruited beyond this point.

Based on the results of Phase I, three open-ended questions (Table 2) were identified and phrased in consultation with two experts in the field. After pre-testing, minor changes (i.e., rephrasing) were made to the list of questions.

**TABLE 2:** Interview questions posed to the participants.

Q1	Describe your day-to-day eating habits
Q2	Have your eating habits or food choices changed since childhood?
Q3	In general, do you see any changes in eating habits and the food environment? If so, what are the reasons for these changes?

#### Data Analysis

The interview recordings were transcribed verbatim, translated to English (wherever necessary) by the interviewer (GK), and thematic analysis was used to analyze the transcripts as explained in Phase I.

## Results

### Phase I (Focus Group Discussions)

Three primary themes including: (i) Conceptualization of processed foods; (ii) Types of processed foods; and (iii) Factors associated with the use of processed foods emerged during the two FGDs.

#### Conceptualization of Processed Foods

Type of processing: The participants perceived processed foods as food products that undergo several chemical and physical treatments in an industrial setting, before they reach the final consumer. Some of these processes included canning, freezing, and pasteurization. The following responses exemplify the concept mentioned above:

“*They* (processed foods) *are food items that has had a series of mechanical or chemical operations performed to change or preserve it*” (P6, F, FGD1).

“…*Definitely, chemicals are used. But I don’t know what chemicals are used. Basically, it is used to prevent items from spoiling*…*It clearly indicates that some sort of processing is happening before it reaches our table”* (P2, F, FGD2).

They believed that the use of additives or chemicals assisted in extending the shelf life of the processed food items. Besides prolonging shelf life, these additives are also added to enhance flavor/color/taste of the processed foods.

*“I assume they* (processed foods) *are foods that include chemicals such as preservatives and other artificial things. These artificial things are sometimes added to processed foods to make their flavor more appealing and to extend their shelf life*…” (P3, F, FGD1).

*“Foods that undergo several industrial processes in which preservatives and other additives are added. They* (processed foods) *are food products with unlimited proportion of taste enhancers. For example, Maggi* (a brand name for noodles) *has got Ajinomoto. It is basically to increase taste*…” (P5, M, FGD2).

Nutritional composition: The high fat, salt, and sugar content of processed foods invited criticism from the participants as their increased consumption may result in obesity and diet-related chronic diseases. They also noted that processed foods had low nutritional value because several nutrients were lost during industrial processing as illustrated by the following quotes:

“…*I am not sure which foods are processed but I think that so much processing can remove all the vitamins. I don’t think they* (processed foods) *are a good thing”* (P7, F, FGD2).

*“I feel they are chemically processed foods, also called ultra-processed foods, tend to be high in sugar, artificial ingredients, refined carbohydrates, etc. Because of this, they are a major contributor to obesity and illness around the world. They usually contain ingredients that could be harmful if consumed in excess, such as saturated fats, added sugar, and salt”* (P7, M, FGD1).

“…*Also, processed food has been highly modified with lots of added calories, including saturated fat, sugar, and starches which has helped fuel the obesity epidemic”* (P2, M, FGD1).

#### Types of Processed Foods

When participants were asked to list processed foods, they listed several ultra-processed foods (e.g., potato chips, bottled juices, cookies, and cakes) and a few items from Group 1 (unprocessed or minimally processed foods) and Group 2 (processed culinary ingredients) of NOVA classification (37).

A variety of reasons for the use of processed foods were mentioned during discussions, which included contemporary socio-cultural as well as economic changes. Based on these results, our next study was designed to further explore the reasons for changes in food choice using semi-structured interviews.

### Phase II (Semi-Structured Interviews)

During the interviews, two primary sets of themes emerged: (i) Changing socio-cultural environment and (ii) Changing food environment which are discussed below. It must be noted that these themes discussed below were also mentioned during the two FGDs.

#### Socio-Demographic Characteristics of the Sample

A total of 22 informants (9 males and 13 females) participated in the face-to-face interviews. All the interviewees were college graduates. Except one, all the participants had an annual household income of 360,000–500,000 (Table 3) and are classified as middle class (50).

**TABLE 3:** Socio-demographic characteristics of the sample (Phase I and Phase II).

		FGDs (*N* = 16)	Interviews (*N* = 22)
Gender	Male	6	9
	Female	10	13
Age (years)	40–50	13	15
	51–65	3	7
Place of residence	Kochi	7	9
	Mumbai	9	13
Education	Graduate	6	12
	Postgraduate and above	10	10
Marital status	Married	13	19
	Divorced	1	0
	Widowed	2	3
Annual household income (Approx.)	200,000–350,000	3	1
	360,000–500,000	13	21
Occupation	Housewife	0	4
	Service	16	12
	Business	0	2
	Retired	0	4

#### Daily Diet and Changes in Diet

In response to the question regarding their daily diet, participants reported eating three meals, of which lunch and dinner consisted of traditional Indian cuisine. Breakfast was the only meal which had incorporated western elements such as bread and jam as well as breakfast cereals such as oats. The residents from Kochi reported replacing rice with rotis at dinner time (Indian flat bread made with whole wheat flour; rotis are the staple diet of North Indians). Eating out or ordering food was largely restricted to the weekends.

“…*Then for breakfast I have oats and milk*… *Heavy lunch in the afternoon with all vegetables included. Wheat products in the night* (chapatti or so)…” (P5, F, KOC).

“…*Breakfast menu itself changed. We started having bread and jam. That did not exist before. Eating out has increased. We also go out and eat. But occasionally, once in a month or so”* (P8, F, KOC).

“…*Normal Kerala style lunch with non-veg. For dinners, we mostly have chappatis* (another name for rotis). *Earlier we used to eat rice all three times*…” (P9, F, KOC).

*“Sugar consumption was lower before, we used jaggery instead”* (P3, F, KOC).

#### Changing Socio-Cultural Environment

Globalization and urbanization: The two terms “globalization” and “urbanization” repeatedly surfaced during the course of interviews and FGDs. These societal changes were held accountable for the transformation of Indian diets. The establishment of large-scale food processing industries and constant influx of imported food products has contributed to tremendous increases in both demand and consumption. This evolving dietary pattern is clearly reflected in the increased consumption of processed and ready-to-eat/heat foods, particularly among the urban middle-class Indian residents.

“…*World is progressing so fast*… *Migration has taken place. Industrialization happened. Have you heard of Paneer Butter Masala before? We never thought that these foods will come to our place”* (P7, M, KOC).

*“Food choice over the world has undergone number of changes over the past few years. Rapid economic growth and globalization are the main drivers for these changes”* (P9, F, MUM).

*“The factors like availability of foreign food products, job of women, urbanization, etc., play a role in the increased consumption of processed foods*…” (P5, M, FGD2).

*“Food packaging has changed, it is all plastic now, it was paper before”* (P3, F, KOC).

Long work days and sedentary lifestyles: Globalization created new job opportunities in urban areas. However, these emerging opportunities invited a lot of criticism as they were associated with long work hours and inflexible work schedules leaving many people feeling short of time and stressed. Subsequently, it resulted in poor eating habits like skipping meals, eating while working, dining out, and eating in the car.

*“With busy schedule and work life, eating out has been the easiest way to reach out to hunger”* (P3, F, FGD1).

“…*Our routine has become faster. You can eat and work simultaneously if you are choosing burger or pizza. It is actually time saving!”* (P1, M, KOC).

“…*Now the nature of work has changed. Especially after the emergence of IT field. You eat whenever you feel like eating and get time”* (P4, F, MUM).

*“Due to stressful and busy work, you’ve almost forgotten you ate lunch and you’re already grabbing something else and eating while working*…” (P12, M, MUM).

Besides poor eating habits, participants also associated the hectic work schedule with reduced frequency of recreational physical activity among the officer-goers.

“…*But they don’t have time to do any physical activity. A complete sedentary lifestyle exists”* (P4, F, FGD2).

Rising income levels: With the advent of new job opportunities, an overall rise in the income levels was reported which in turn had a significant impact on food choices. Participants indicated that because of this increased purchasing power many consumers are willing to spend more on food. Undoubtedly, this has resulted in increased frequency of dining out and buying of processed foods among the urban population as reported by our sample.

The following quotes explain the impact of rising income levels:

*“Increase of per capita incomes and changes in lifestyle have changed our food preferences*…*Now-a-days an increasing number of young people have higher disposable in-comes than their older counterparts and have a tendency to spend their money on ready-to-eat foods*…” (P12, M, MUM).

“…*We did not have enough money before. Earlier we did not have money to buy it. It is all about money. They are ready to spend”* (P2, M, FGD1).

*“Now, people earn a lot compared to earlier. So, they are able to buy anything they want*…” (P5, M, KOC).

Decline in household cooking (Women in the workforce and Change in household structure associated with lack of time): Unanimously, all our study participants claimed that there has been a gradual decline in household cooking in urban households. This decline was partly attributed to increased participation of women in the paid workforce and emergence of dual-income families. The participants felt that employment duties make it challenging for women to cook as meal planning and food preparation are time consuming activities. Consequently, this resulted in reliance on ready-to-eat/heat meals and increase in dining out episodes as cited by the participants.

*“Both male and female members of the family are now involved in nine to six jobs where they hardly get time to cook for themselves”* (P8, F, MUM).

*“In the previous generation, women thought that they have to take care of their family by feeding them properly. Now that is not the case”* (P9, M, KOC).

*“Parents have to nourish healthy habits in children, they have to make an effort to cook delicious food at home”* (P7, M, KOC).

Another reason for the waning household culinary practices was change in household structure. Participants believed that the current generation missed learning various traditional recipes as the traditional joint family system was replaced by nuclear families. They further disclosed that cooking for family functions and festivals is slowly disappearing as it requires too much effort and time. With regard to this, either the food is catered, or people celebrate festivals in restaurants.

*“We know that working women and reduction in the size of families are also contributing to food choice changes”* (P4, M, KOC).

*“Since there are very few members in the family now, if we buy ready-made snacks that would be enough for all family members for many days and lessens the effort*…” (P3, F, FGD1).

*“We are surrounded by a number of celebrations. None of us have time to cook and serve on this occasion. Hence, the choice is to buy such ready-made products”* (P12, M, MUM).

#### Changing Food Environment

Food diversity: A large segment of the sample mentioned the replacement of traditional Indian diet with elements of Western diet and exposure to different cuisines. This food diversity was attributed to migration, growth of food industry, export and import of food items, advertising, high purchasing power, and establishment of global restaurants and outlets. The following statements are suggestive of the on-going food diversification:

“*We are actually shifting toward foreign culture by leaving aside our own culture and food. There are many catering services now. We were not aware of these varieties of food earlier. We look into internet and learn new recipes*…” (P2, F, KOC).

“…. *Once migration happened, people started coming from different places. This has led to emergence of multiple cuisines*…” (P4, F, MUM).

*“We witnessed a proliferation of fine dining restaurants, cafes, pubs, bars, clubs, lounges, and international fast food joints in recent times*… *One of the real game changers was the entry of the American food chains such as McDonald’s, KFC, Domino’s Pizza, Pizza Hut, etc. This can be viewed as a result of the fast-growing economy, industrialization, constant travel across the world*…” (P8, F, MUM).

Availability and Accessibility: Participants cited that increased availability and easy access of unhealthy food at home or outside makes people inclined toward eating these foods. They also mentioned that accessibility to shops is another important factor influencing food choice, which is dependent on resources such as transport and geographical location. Globalization has played a vital role in it. The following statements exemplify this concept:

*“I can buy anything I want, as everything is available in market”* (P1, M, MUM).

*“Now-a-days all food products are available everywhere, people can come and eat without anybody’s help, transportation is easy*…” (P3, F, KOC).

*“Earlier, it* (unhealthy food) *was available only in town/city. Now you get it everywhere and there are hundreds of shops open in our native place too”* (P2, F, FGD2).

Convenience: Participants stated that people value convenience so highly that they are ready to spend a lot on food that requires little or no preparation, as well as time saving appliances and easy to clean cookware.

*“Increase in numbers of convenience foods and prepared meals available is the prime reason for changing food choices”* (P2, M, FGD1).

*“A lot of household appliances are used now, ready to use dosa (savory crepes) batter is bought”* (earlier it was hand ground at home on a grinding stone) (P4, F, KOC).

*“Now people make large quantities and store in the fridge. We never used fridge before, everything was made fresh*…*Cooking utensils are different, earlier we used earthen utensils not non-stick pans”* (P3, F, KOC).

*“The modern supermarkets are offering variety of food products which are more convenient to ease the lifestyle of people as compared to the traditional options”* (P3, M, MUM).

Advertising and media: Our study sample felt that mass media is dominated by advertisements of ultra-processed foods and sugar-sweetened beverages. Unfortunately, these attractive food advertisements are mostly targeted toward children and have been successful in luring children as well as influencing their food choices and preferences. This in turn left a strong impact on the primary food gatekeeper (e.g., mother) as noted by our interviewees and FGD participants.

*“TV advertisements are undoubtedly the biggest and single largest factor responsible for change in eating habits”* (P12, M, MUM).

*“Addiction to eating junk food has increased by way of advertisement”* (P2, F, KOC).

*“Advertisements are very attractive. Children fall into the trap of these advertisements”* (P7, F, FGD2).

*“What I buy or eat is what they (children) like and demand*…” (P8, F, MUM).

*“Children do not like traditional food. They always fall for hotel food and junk food”* (P1, F, KOC).

On the other hand, the respondents also discussed the positive impact of media on food choices. This was seen in the rapid movement of cookbooks and cooking shows that are increasingly focusing on traditional recipes.

Food as identity: During the focus groups and interviews, food emerged as a platform for expressing identity which was associated with a social purpose. In the urban way of life, people used food to define themselves in terms of religion, ethnicity, social class, and so on. Hence, eating a Westernized diet and not a traditional Indian diet is reflective of one’s identity. Participants frequently mentioned that people derive status from consuming “western foods” (e.g., pizza, burger) or dining at fast food chains and restaurants. They also added that purchasing organic food or being brand conscious reflected privilege and status.

*“We are going toward western food diet and culture. We feel that is prestigious”* (P7, F, MUM).

*“Social status takes people in an unending vicious circle wherein he is constantly comparing his social status with respect to food choice and the choice of restaurants”* (P2, F, KOC).

“…*We became brand conscious. Earlier, we used to grind all powders (spices). Now we are buying branded instant powders”* (P1, M, MUM).

“*Sometimes people also prefer dining out just to flaunt their social quotient*…” (P13, F, MUM).

*“Status and prestige play a major role in that. We need that social status, or else we feel inferior. In order to reach that standard, we go out and eat”* (P4, F, FGD2).

Lifestyle Diseases and Quality of food: The burgeoning fast food environment and sedentary lifestyles were often held responsible for the mounting prevalence of NCDs. The following responses exemplify this detrimental health impact:

“*Disordered food choice can lead to metabolic diseases and even cancer*” (P1, M, MUM).

“*Lack of exercise and outside food would lead to many diseases. People go for mall culture. Consumerism has made a lot of changes in our food habits and choices*…*There is lot of health implication for this change. Due to this many chronic diseases are on the rise”* (P3, F, KOC).

*“I feel they are chemically processed foods, also called ultra-processed foods, tend to be high in sugar, artificial ingredients, refined carbohydrates, etc. Because of this, they are a major contributor to obesity and illness around the world”* (P7, M, FGD1).

Participants further expressed their concern over the rampant use of pesticides and fertilizers. The decline in the availability of fresh and minimally processed foods and changing nutrient quality also received criticism from several participants.

*“Usage of pesticides and other chemicals not only reduces the quality of fruits and vegetables but also pose potential risk to health”* (P2, F, KOC).

“…*It* (processing) *removes some of the nutrients and vitamins. They are obtained from lab not nature. They are genetically modified”* (P3, F, FGD1).

*“All masala* (spice) *powders have changed. Earlier, we used to grind and make powders. We get everything in ready-made packets”* (P3, F, KOC).

## Discussion

This study focused on participants’ understanding of processed food as well as descriptions of their daily diet and changes in food choice. The reasons for changes in food choice behavior have also been explored. Their understanding of processed food was based largely on the category of ultra- processed items that came to mind more easily such as, noodles, instant soups, chocolates, and confectionery that are advertised regularly. Participants did not differentiate between processed and ultra-processed as a category although they did list culinary ingredients such as oil and spices in a separate category. Interestingly, bread and cheese were not listed as processed foods. They discussed the high calorie content and high levels of sugar, salt, refined carbohydrates, saturated fat, and artificial ingredients present in processed foods and its negative impact on health.

In terms of the reasons for changes in food choice behavior, globalization, urbanization and increased income were repeatedly mentioned as key factors that played a role through changes in the physical, social, psychological and cultural environment. Physical aspects refer to the actual built up, post-industrial environment of cities, social aspects refer to the new workplace and its culture, the psychological aspects refer to attitudes, habits and self-regulation and cultural aspects refer to changing values. Globalization is the overarching transformation that subsumes many changes such as migration, engagement of women in the paid workforce, and change in traditional culture and cuisine which are discussed in detail.

Globalization has brought in its wake multinational firms (59) and a work culture that involves long hours (60). The information technology (IT) industry which is a large part of the Indian economy has long and erratic hours (61) and thus the hours spent at work have increased significantly resulting in a sedentary lifestyle (62, 63). Employment in the IT industry for men and women has changed the power equation among couples with women spending less time in the kitchen. Our results also revealed that IT employees often snacked on processed food at work.

Indians have until recently, been rather rigid about their food choices with caste and region playing a determining role in their diet (33). However, the winds of economic change have gradually changed their outlook toward food (12). Increased income levels have led to an openness towards new food as well as eating out and ordering in (59) as mentioned by a majority of the participants. People are now trying foreign brands and new products such as foreign cheese and olive oil. Even, the breakfast cereal market in India has been steadily growing and in the south of India, oats have become very popular (64); a finding also echoed in our study.

In earlier times cooking was the chief occupation of women and cooking for large joint families was a gargantuan task as reported by women participants. A lot of food processing took place at home such as pickling, drying vegetables, making papads (dried chips), spice powders and mixes which was done on a seasonal basis. With urbanization and apartment style housing and rapidly shrinking kitchens, the approach to cooking changed. Nuclear families (65, 66) and women going out to work (60, 67) have further altered the dynamics of home cooking (42). Nonetheless, Indian women still do a lot of the cooking during the week using time saving cooking techniques (68). Participants also mentioned now using readymade spice powders, pickles and papads as a convenience. Tea-time snacks are another category that is most often store bought rather than home-made as was done previously. Indeed, Law et al. reported a considerable rise in the purchase of sweet and salty snacks in urban India between 2013 and 2017 (16). In India numerous festivals are celebrated with the extended family getting together and enjoying elaborate meals which involve tremendous time and effort. The study participants observed that a lot of these festival foods are now commercially available, or family events are either catered or the entire family eats at a restaurant. The attitude toward cooking has changed even among homemakers who view it as one part of their responsibility that has to be addressed efficiently. Cooking no longer contributes to a woman’s identity in a central way anymore.

This change in eating habits and cooking patterns observed by the study participants maps onto the fourth stage of the “nutrition transition” as outlined by Drewnowski and Popkin (22). In modern urban society, energy needs are low, however, the values governing food and eating still stay rooted in an earlier stage and link quantity and abundance with prosperity and celebration. There is a lag in the transformation of value systems to fit with contemporary energy needs, which is referred to as the obesity transition (69). This transition is related to socio-economic position as energy needs change. In India the middle class is probably in the fourth stage of the nutrition transition, evidenced by the rising NCD level (48, 70). Among other countries transitioning economically and socially like India, Nepal a neighboring south Asian country also reported being in the fourth stage of the nutrition transition (71). However, some participants mentioned that among the highly educated upper income category, an effort is being made to go back to an older traditional diet, indicating a transition to the fifth stage. Indeed, there is a revival of the traditional diet rich in millets with an emphasis on organic food among the educated elite (72, 73).

The Indian food industry is expanding rapidly and the food processing sector accounts for 32% of it (74). The federal government’s food policy is actively encouraging food processing and foreign investment in India which has boosted the availability and affordability of processed foods (75). Foreign direct investment inflows worth USD 904.70 million from global investors was received in the year 2019–2020, a 44% increase from the previous year (76). Thus, the food environment in India is gradually changing and there is a plethora of Indian and foreign foods available to the consumer. The presence of western food and culture was also referred to as one of the factors changing the food environment, not just in terms of food but also in terms of values of eating out and eating pre-prepared foods. Sometimes, once a week or so, foreign cuisines maybe prepared as a change from the usual menu. Middle eastern, Italian, Mexican and Chinese cuisines are popular among the middle class in the metro cities where ingredients for these cuisines are easily available. Furthermore, migration to middle eastern countries, United Kingdom and United States (77), has familiarized the local population with foreign brands. People working abroad return home with foreign foods and beverages for their families as gifts and gradually people acquire a taste for these brands which are now available in the local markets such as Tang (orange beverage powder), chocolate brands such as Lindt and Quality Street (mentioned by the participants in Kerala). The arrival of international fast food chains such as Pizza Hut, McDonalds, KFC are referred to as a “game changer” by one of the participants in the context of the explosion of restaurants, pubs, cafes, etc. Fast food chains apart from being perceived as “trendy” and western, are also hygienic, provide value for money and a space for young people to “hang out” thus leading them to identify with specific fast food chains.

The availability of processed foods not just at supermarkets but also at local grocery stores have led to their increased consumption. One participant mentioned that shops were everywhere, so anything that they thought of, they could easily buy. And now with everything being delivered, things are merely a swipe away juxtaposed against earlier times when grocery lists were made, and weekly or monthly shopping trips were planned. Well stocked shops and neighborhood cafes prime people to be engaged with food, cooking, and eating (48). Research indicates that food imagery has a significant effect as a prime for overeating (78), in both adults and children (79).

The internet, is another medium through which international cuisine has spread, as mentioned by participants. Cooking shows aired on YouTube and television showcase both Indian as well as foreign cuisines. Fascinatingly, each Indian state has its unique regional cuisine and for people to try cuisines from other states is a big departure from their traditional cuisine. Hence one of our study participants from Kerala (Kochi) which is one of the southernmost states mentioned that “Have you heard of Paneer Butter Masala before? We never thought that these foods will come to our place…”. Paneer Butter Masala is a cottage cheese dish from Punjab in the north. Paneer or cottage cheese is never used in south Indian cuisine and hence is almost foreign to older people. Participants also reported TV viewing to be associated with increased consumption of unhealthy foods, specifically processed food and increased requests of foods seen in advertisements by children. These views are consistent with research that indicates that where there is high volume of child centric advertising of processed foods and children indicate a preference for these advertised foods (41, 80, 81). Children’s exposure to advertising is considered a significant factor contributing to their unhealthy diet in the United States (82, 83) as well as in India (41, 42). Nonetheless, a positive aspect of the media acknowledged by some participants was that of the internet and cookbooks which showcased traditional recipes that helped in revitalizing classic traditional cuisines, a finding often reported in the last decade (41, 68, 84–86).

Eating out as an indicator of status was mentioned by many participants. Participants mentioned that preferring western cuisine was seen as a marker of identity and status. The entry of many foreign cuisines is a new phenomenon (87) and people are enjoying the novelty and variety of dining options now available. Eating out was a rare treat for the middle class but now with the dinning environment changing (13, 48, 87) and increased income, eating out frequently has become popular. Ordering food has also become very frequent and is more popular than eating out (88). The online ordering market in the metros has grown steeply and is worth more than 5 billion USD and is expected to keep growing (89). Thus, the culture of eating out appears to be firmly established and growing. However, this trend of eating out and processed food consumption comes at a cost.

The connection between processed food consumption and NCDs was pointed out by participants. They mentioned that lack of exercise and reduced home-made food intake were the major factors responsible for NCDs. The high levels of sugar and refined carbohydrates in processed foods were highlighted as being harmful, thus indicating their awareness of the link between processed food and NCDs. Changing food habits and increased working hours leading to a more sedentary lifestyle are responsible for the rise in NCDs in India (12, 14, 48, 90). Participants also expressed concern over food being contaminated by fertilizer and pesticide. They mentioned that fruits and vegetables were depleted of their vitamins and their nutritive content was not the same anymore. This heightened awareness of NCDs and fertilizers within our sample could be attributed to the fact that all our participants had a university degree. Fernandes (91) described the post-liberalization middle class as a social group, typically comprises English-speaking cosmopolitan white-collar workers (91). Moreover, Kerala has the highest literacy rate in India (94%) and even Maharashtra’s literacy rate (82%) is much higher than the national average, i.e., 74% (92). This could also partially validate the nutritional awareness among the interviewees and the FGD participants. Nevertheless, this level of nutritional awareness may not be prevalent among other socio-economic classes like the lower middle or the lower income group and in different geographical settings.

The limitations of this study should be acknowledged when interpreting the study findings. First, the study sample may not be representative of the urban Indian population because the selection of participants was limited to only two cities. Nevertheless, it should be noted that Mumbai is a cosmopolitan city which hosts people from different Indian states. Moreover, qualitative inquiries are designed to obtain deep insights rather than focusing on generalization of findings. Second, social desirability could have biased responses. Third, the dominance of women in the FGDs and interviews may represent a source of gender bias in the present inquiry.

Despite these limitations, this study also had strengths. It is one of the first qualitative studies to be conducted in India exploring the urban, middle-class consumers’ views about processed foods and the factors affecting evolving food choices. Another strength of our study, is the unanimous views of both Kochi and Mumbai residents regarding processed foods and evolving dietary choices, highlighting the on-going nutrition transition and need for effective nutrition interventions and public health policies.

The results illustrate how market forces and culture interact to influence individual behavior as well as the overall food environment which subsequently affects the health of people. Many participants are aware of the unhealthy aspects of processed food, however, considering the growing market share of these foods it is obvious that not everyone is aware, and the convenience and novelty value of certain foods override the health concerns. Interventions to improve the nutritional knowledge of young people and parents of young children would be of value in helping people make healthier choices.

Nutrition education regarding the link between processed food and NCDs and the lower energy needs of urban people will enable people to make better choices. The importance of buying local fresh produce must be emphasized as well as the importance of retaining culinary traditions despite new influences. Nutrition education equips consumers to make informed decisions regarding the nutritive value of new products as well as cuisines and improves their self-efficacy regarding planning and preparing a varied and healthy menu. The value of home-made food must be highlighted as well as the opportunity cost of women’s time. If food related tasks are shared in an egalitarian manner within the family it would make traditional cuisine more sustainable, thereby promoting healthy eating among urban Indian families.

## Data Availability Statement

The raw data supporting the conclusions of this article will be made available by the authors, without undue reservation.

## Ethics Statement

The research protocol for this qualitative investigation (i.e., both Phase I and Phase II) was approved by the Institutional Ethics Committee of the Indian Institute of Technology, Bombay (IITB-IEC/2019/029). The participants provided their written informed consent to participate in this study.

## Author Contributions

GK and MK conceived the study and its original design. GK collected the data. GK, NR, and MK analyzed the data, drafted the initial form and all revisions of the manuscript, and reviewed and approved the final manuscript. All authors contributed to the article and approved the submitted version.

## Conflict of Interest

The authors declare that the research was conducted in the absence of any commercial or financial relationships that could be construed as a potential conflict of interest.

## Publisher’s Note

All claims expressed in this article are solely those of the authors and do not necessarily represent those of their affiliated organizations, or those of the publisher, the editors and the reviewers. Any product that may be evaluated in this article, or claim that may be made by its manufacturer, is not guaranteed or endorsed by the publisher.
